# An Address Delivered at the Last Meeting of the Medical Association of Georgia

**Published:** 1867-04

**Authors:** J. T. Banks

**Affiliations:** Chair


ATLANTA

### Medical and Surgical Journal.

NEW SERIES.

VOL. VIII. APRIL, 1867. No. 2.

### ORIGINAL COMMUNICATIONS.

### ARTICLE I.

*An Address delivered at the last meeting of the Medical Association of Georgia.* By the late President, Dr. J. T. BANKS, on retiring from the Chair. (Furnished for publication by request of the Association.)

*Gentlemen of the Medical Association of Georgia.*

The time has arrived for me to return to you my sincere thanks for the honor you bestowed upon me at our last meeting, in electing me your presiding officer. This honor I have never regarded in a selfish light: it was not bestowed because of any special excellence or merit, but as a reward for my professional devotion, and my constancy to the interest of this Association.

In 1860, this Association held its annual meeting in the City of Rome, and bestowed its highest honor on our much esteemed and able brother, Dr. Hayden Coe, who, in the wisdom of God, has been taken from our Association, and placed beyond the reach of earth's fading honors, to reap his endless reward at the bar of the high Court of Jehovah. In our Association his death is painfully felt, having arrived at that period in life, when long years of labor and study were fast ripening into lessons of wisdom.

Then, Georgia was in the sisterhood of States that formed the once glorious American Union, and our Association annually represented in the American Medical Association. But, ere we met again, a combination of causes, grievous and unendurable, and dating far back in our history for origin, had culminated in, as we most earnestly desired and believed, a final separation. But, alas, how fallacious were our calculations! naught but problems in error awaiting the cycle of time for solution.

When last we met, it was in the beginning of a new era—one in which we painted ourselves in scenes of peace and happiness, in a harvest of plenty, in a confederacy of States independent and prosperous, and able to bear our *great sin of slavery*, over which puritanical New England had been weeping so long because of association. We saw ourselves in a prosperous future, seated beneath our own vine and fig tree, where none dared molest, or make us afraid. In the language of an imprisoned Confederate soldier,

> "Fair as were our visions! Oh they were as grand
> As ever floated out of Fancy land;
> Children we in single faith,—
> But God-like children, whom, nor death,
> Nor threat, nor danger drove from honor's path."

For four long eventful and historic years, we carried the olive branch in one hand, and the sword in the other. Forced to fight for our existence and independence, after making honorable overtures, and asking to be let alone to bear our own burden, and to reap the rewards of our own labor, I now feel a clear conscience in the sight of God, that the blood of millions cry not aloud at our door.

As I before remarked, our last meeting was in the beginning of a new era. Georgia, in her sovereign capacity, had, for good and sufficient cause, withdrawn her voluntary copartnership with the Federal government, and with other sister States that had acted likewise, formed for themselves a new government. Under its ægis, our Association, by resolution, invited the State Medical Organizations within the limits of the Confederate States, to consider with us the propriety of organizing a Southern Medical Association; but ere this invitation could be acted upon, or this Association meet again, our fair land was deluged in blood, and our professional services called from their peaceful labors in the fields of science, to the more dazzling work of the gory field.

But may it be that the God of War has drank to fullness of the gory bowl, that peace may spread her nestling wing again over our oppressed and ruined people, guarding and protecting them against the ruthless hand of a fanatical tyranny they so earnestly sought to escape, and revivify the feeble germ of liberty, life, and hope, that it may again grow in prosperty and power! We must again buckle on our armour, and, as honorable contestants, enter the fields of Medical science. These fields are daily extending as science progresses, and are equally inviting and productive now as when Sydenham, the great father of English Medicine, labored therein. They are the same fields that the jewels of our profession have labored in, from the dawn of science until the present time. Let us, with the same self-sacrificing industry, continue the good work. Let wisdom, guided by observation, handle the pruning knife. Let the bold experimenter engraft in fruitless trees the improvements and the inventions of genius; and let philosophic investigation and inductive reasoning fertilize the soil.

To do this work, we must have harmony and concert of action. To have these, requires the *faithful performance of our duties* to our patients, our profession, and the public. Perform these duties, dismantled of selfishness, in a spirit of liberality, governed by the principles of honor, and actuated by a proper appreciation of our profession, and no pen can write our merited eulogy. These duties are plainly taught in our Code of Ethics, and every member of this Association is pledged in honor to conform to these rules, as far as possible. To be ignorant of them, or to violate them for selfish purposes, is to trifle with our plighted honor. I am aware, that, in our present state of Medical Science, and the ignorance of the public of our professional acquirements, an exact technical conformity with the demands of our Code is impossible. But imperfection is no license to error: it should serve as a healthy stimulus in our efforts to attain a higher standard of professional excellence.

The study of our cases, for their professional interest, is evidence to all observers of an honesty of purpose, self-reliance in our profession, and a sincere desire for its advancement upon true scientific principles. It will elevate it in the estimation of observers above the paltry consideration of dollars and cents, showing one significant and no less commendable difference between the *isms* and deceptions of the day, and true legitimate medicine.

"There is no profession, from the members of which greater purity of character and a higher standard of moral excellence are required than the medical." To attain the highest standard of moral and professional excellence attainable, should be the ambition of every doctor. So often have we heard our profession charged with corruption, and though satisfied that this charge is in the main without foundation, yet I am pained to admit that it is sometimes true. The cry of disease and danger are sounded in the ear of the confiding: a system of medication is at once adopted, long, tedious, and confining, only to terminate when an enormous bill can be plausibly exacted.

Such thievish malevolence can not be too severly punished, and when detected, should be made an object of public scorn. But we should be slow in believing the many reports and accusations against our professional brothers that come wafted on the breeze of public gossip. Many, very many, of them are false; they would pull down the reputation of one, while they endeavor to flatter the vanity of another, that the spawner of the lie may profit by it. Over them let us spread the mantle of charity; pity, rather than censure them: they know not what they do. Daily they refuse the services of honest scientific physicians, and encourage by their patronage the veriest quacks and boastful pretenders, until taught by piercing pain, and empty purses, in the school of experience, their sad mistake.

Of the scientific claims of physicians, it is unfortunate that the people, the party most interested, are, in the main, incompetent to decide; and therefore it is of vital importance to them that our profession should be well informed, and *honest* in practice. But say many, you doctors differ so much we can not tell who is right, or who is wrong! If scientific doctors differ, so do lawyers, divines, statesmen, warriors, civilians—all! Doctors disagree, and in the very nature of things must continue to do so, as long as it is the lot of man to work out his own temporal destiny. When the diversified interest of man is harmonized—when the bond of brotherly love shall enclose the family of man with our interest and our destiny—then, and not till then, will these differences be no more. But should *scientific medicine* be rejected because doctors differ? If so, reject the gathered harvest because *agriculturists* differ; reject all forms of government because statesmen differ; yea! reject the Bible, the revealed will of God, because divines differ; reject it because more than a thousand different religious sects found their faith in its teachings—each claiming for itself the high pre rogative of being the only true church.

But how is true scientific medicine to be known? As a tree is known by its fruit, so is true scientifiic medicine known by the results of its labor. As a general rule is recognized and strengthened by the few exceptions which present its universal application, so is the tree of true scientific medicine known by the puny off-shoots of its own defects. It is known by the field in which its labors are found. It is not bounded by the limits of the vegetable or mineral kingdoms, but is co-extensive with the explorations of science. It is not limited to the exclusive use of water, either cold or hot. Its devotees worship at the shrine of no theoretical dogma, no "*similia similebus curantur;*" nor the opposite of "*contraria contrarius curantur*" can fix a boundery to its labor, or limit its usefulness. It is known as the heir of the accumulated medical knowledge of all nations. And lastly, it is known by the simple yet ancient title of its followers; viz, *Doctors.* Here let me caution our thoughtless brothers against the disingenuous effort being made by the isms and pathies of the day, to force upon us an additional qualifying title. This is the more necessary because a few ignorant or inadvertent admissions have aided our enemies in directing the public mind into the error, that we, too, practice a system of exclusivism. That because a few became prejudiced against some potent mineral medicine, (resulting from misapplication, or abusive use) and founded a system of medication limited to the use of vegetables, and calling themselves Botanics, we must of course, be distinguished, be called mineral doctors, and be opposed to the use of their more favorite remedies; or, because a few, calling themselves Hydropathics, and limiting their medical information and application to the exclusive use of water, that we must be opposed to its use, and ignorant of its application; or, that because the adoption of, fanciful theoretical dogma of the Homeopathist, of like curing like, we of course must adopt the contrary system, and be Allopathist, a term applied to us by the Homeopathist, and now through the improper admission and use of the term by some of our profession, our distinguishing title in the common literature of the country. This is all wrong: it places us in a false light before the public. We are not, and never have been exclusionist. *True scientific medicine knows no other title for its Alumnus but that of Doctor.*

The history of Medicine teaches that for years and years its leading spirits were the advocates of erroneous theories; but the age of inductive philosophy in which we live, has by a different and more correct mode of reasoning, disposed of them, to be known no more but in history. This is an era of scientific progress—an era in which the science of treatment of diseases has made more progress than any of the so-called learned professions. This can be the better understood, when it is known that the rapid improvements and developments in Natural science tend more directly to the advancement of our profession than any other. Physics and Chemistry are now the handmaids of Medicine. Practice, that a few years ago was empirical, is now national and scientific. The Chemical analysis of the fluids of the body, in health and disease, often enables the scientific physician to prescribe, with almost a mathematical certainty, the needful remedies. The closeted Chemist of France, Germany, or of any other government, makes a discovery, and it is at once heralded to the limits of civilization, and applied by our prefession for the benefit of all. Scientific Medicine is the common property of the Medical students of all nations. As our government advances in medical lore, so is the pathway of Medicine illuminated in every other. Our *body medical* extending to the limits of civilization, and having, and tolerating no medical arcana, but laboring as a band of brothers, wherever found, for the same noble and philanthropic purpose, gives us, by this united and coöperative labor, a great advantage in progress over other professions, and makes it, to every thoughtful observer, an object of admiration.

This Association has been called, that we may again labor together in the fields of science, and continue to elevate the science of Medicine in the estimation and admiration of the observing public. Our annual meetings afford us recreation from our arduous labor, and are alike convivial and instructive. Here, each one adds his mite on the altar of our profession for the common good. Here, we are, by association, merged into the profession, making our action the action of the profession. Then let us smooth down the asperities which are naturally engendered, even in honorable competition, and in our action know nothing but our profession, its interestand advancement.

In addition to Essays, Reports, and Communications, a few important subjects should engage our attention at this meeting. The new feature adopted at our last meeting, of awarding prizes for Essays, should, in my opinion, be continued. The importance of a law requiring and regulating the registration of births, vaccination, marriage, and deaths, should continue to be urged upon the consideration of our law-making power. These subjects require no argument at my hands to enlist your interest.

The propriety of locating our Association at some convenient, accessible, and commodious place, is a question that was postponed for further consideration at our last meeting, and claims your attention at this. Let us meet this subject in a spirit of fairness, liberality, and compromise, looking alone to the interest of the Association. In my opinion, itis of paramount importance to the Association that it be located. If you wish to build up a professional reputation for Georgia, this is the channel in which to labor. It will soon inspirit a commendable Medico-State pride that will gather together an extensive Library and interesting Museum. There we can deposit our private collections without favoritism, and feel that we are not only adding to, and advancing the claims and usefulness of our science, but we are also recording our names in the history of Medicine, to live in praise of our industry and professional devotion when all else may be forgotten in death.

In conclusion, permit me to commend to your faithful care the interest of our Association, and through it our *noble profession.* Noble, because of our exalted mission. It is in the fulfillment of our mission—the highest and noblest work of man—that man, made in the image of his God, approximates nearest the attributes of a God. What picture of business life can excell that of the true, faithful, scientific physician? View him in faithful performance of his duties, countless of cost, and regardless of danger! Disease may palsy his limbs and pale his cheeks; but fear never.

